# Supplementary material for: Genetic and molecular characterization of multicomponent resistance of Pseudomonas against allicin
Source: Life Sci Alliance. 2020 Mar 31;3(5):e202000670. doi: 10.26508/lsa.202000670 (PMC7119367; doi:10.26508/lsa.202000670)
Supplement: Supplementary file 1 [file LSA-2020-00670_TableS1.docx]

**Table S1**: Genes from the three genomic repeats RE1, RE2, and RE3 located in the *Pf*AR-1 genome, related to section “*In-silico analysis of the* Pf*AR-1 genome”*. The congruent gene set for all three repeats is marked in green, the congruent set for only two of the repeats is marked in orange, unique features for a repeat are marked in grey.

| **gene identifier** | | | **% amino acid identity** | | |  |  |
| --- | --- | --- | --- | --- | --- | --- | --- |
| **RE1** | **RE2** | **RE3** | **RE1 vs RE2** | **RE1 vs RE3** | **R2 vs RE3** | **RAST annotation** | **RE core annotation** |
| PfAR1.g10570 | PfAR1.g25760 | PfAR1.g36550 | 97.91 | 67.03 | 68.11 | Transcriptional regulator, TetR family | *tetR* |
| PfAR1.g10610 | PfAR1.g25790 | PfAR1.g36540 | 98.91 | 89.67 | 89.67 | OsmC family protein | *osmC* |
| PfAR1.g10620 | PfAR1.g25800 | PfAR1.g36530 | 97.67 | 91.05 | 91.83 | Short-chain dehydrogenase/reductase SDR | *sdr* |
| PfAR1.g10630 | PfAR1.g25810 | PfAR1.g36520 | 98.98 | 86.73 | 87.76 | Transcriptional regulator, TetR family | *tetR* |
| PfAR1.g10640 | PfAR1.g25820 | PfAR1.g36510 | 96.98 | 77.16 | 75.43 | isomerase, putative | *dsbA* |
| PfAR1.g10650 | PfAR1.g25830 | PfAR1.g36500 | 97.25 | 89.91 | 88.99 | Thiosulfate sulfurtransferase, rhodanese (EC 2.8.1.1) | *trx* |
| PfAR1.g10660 | PfAR1.g25840 | PfAR1.g36490 | 98.52 | 92.98 | 92.98 | Glutathione-regulated potassium-efflux system protein KefC | *kefC* |
| PfAR1.g10665 | PfAR1.g25845 | PfAR1.g36480 | 93.64 | 77.91 | 79.07 | Glutathione-regulated potassium-efflux system ancillary protein KefF | *kefF* |
| PfAR1.g10670 | PfAR1.g25850 | PfAR1.g36470 | 98.61 | 97.22 | 95.83 | 4-oxalocrotonate tautomerase family protein | *4-ot* |
| PfAR1.g10680 | PfAR1.g25860 | PfAR1.g36460 | 99.18 | 94.29 | 94.02 | NADH:flavin oxidoreductases, Old Yellow Enzyme family | *oye* |
| PfAR1.g10690 | PfAR1.g25870 | PfAR1.g36450 | 100.00 | 96.36 | 96.36 | Possible carboxymuconolactone decarboxylase family protein (EC 4.1.1.44) | *ahpD* |
| PfAR1.g10710 | PfAR1.g25880 | PfAR1.g36395 | 91.18 | 87.50 | 89.22 | ethD reductase (originally annotated as hypothetical protein) | *ethD reductase* |
| PfAR1.g10720 | PfAR1.g25890 | PfAR1.g36390 | 98.63 | 91.10 | 90.75 | Alpha-ketoglutarate-dependent taurine dioxygenase (EC 1.14.11.17) | *tauD dioxygenase* |
| PfAR1.g10730 | PfAR1.g25900 | PfAR1.g36380 | 98.50% | 93.23 | 93.05 | Carotenoid cis-trans isomerase (EC 5.2.-.-) | *oxidoreductase* |
| PfAR1.g10740 | PfAR1.g25910 | PfAR1.g36370 | 96.97 | 80.99 | 80.99 | Transcriptional regulator, AraC family | *araC* |
| PfAR1.g10560 | PfAR1.g25755 |  | 90.79 |  |  | Glycine cleavage system transcriptional activator GcvA |  |
| PfAR1.g10560 | PfAR1.g25757 |  | 88.61 |  |  | Glycine cleavage system transcriptional activator GcvA |  |
| PfAR1.g10572 | PfAR1.g25765 |  | 98.10 |  |  | Permease of the drug/metabolite transporter (DMT) superfamily |  |
| PfAR1.g10590 | PfAR1.g25770 |  | 92.54 |  |  | Cystathionine beta-lyase (EC 4.4.1.8) |  |
| PfAR1.g10600 | PfAR1.g25780 |  | 96.90 |  |  | L-asparagine permease |  |
| PfAR1.g10750 | PfAR1.g25940 |  | 98.89 |  |  | Glutathione reductase (EC 1.8.1.7) |  |
| PfAR1.g10755 | PfAR1.g25945 |  | 98.18 |  |  | hypothetical protein |  |
| PfAR1.g10760 | PfAR1.g25950 |  | 99.60 |  |  | Bll2902 |  |
| PfAR1.g10700 |  | PfAR1.g36445 |  | 77.13 |  | Acyl carrier protein phosphodiesterase (EC 3.1.4.14) |  |
| PfAR1.g10574 |  |  |  |  |  | hypothetical protein |  |
| PfAR1.g10576 |  |  |  |  |  | hypothetical protein |  |
| PfAR1.g10578 |  |  |  |  |  | Outer membrane protein W precursor |  |
| PfAR1.g10583 |  |  |  |  |  | Hydrolase, alpha/beta fold family protein |  |
| PfAR1.g10586 |  |  |  |  |  | Transcriptional regulator, TetR family |  |
| PfAR1.g10595 |  |  |  |  |  | hypothetical protein |  |
| PfAR1.g10695 |  |  |  |  |  | hypothetical protein |  |
| PfAR1.g10580 |  |  |  |  |  | probable short-chain dehydrogenase |  |
|  | PfAR1.g25752 |  |  |  |  | hypothetical protein |  |
|  | PfAR1.g25775 |  |  |  |  | hypothetical protein |  |
|  | PfAR1.g25785 |  |  |  |  | hypothetical protein |  |
|  | PfAR1.g25920 |  |  |  |  | Transcriptional regulator, LysR family |  |
|  | PfAR1.g25930 |  |  |  |  | putative aldo/keto reductase |  |
|  |  | PfAR1.g36545 |  |  |  | hypothetical protein |  |
|  |  | PfAR1.g36440 |  |  |  | Transcriptional regulator, AsnC family |  |
|  |  | PfAR1.g36435 |  |  |  | Cyclohexadienyl dehydratase (EC 4.2.1.51)(EC 4.2.1.91) # Periplasmic precursor |  |
|  |  | PfAR1.g36430 |  |  |  | amino acid ABC transporter, ATP-binding protein |  |
|  |  | PfAR1.g36426 |  |  |  | polar amino acid ABC transporter, inner membrane subunit |  |
|  |  | PfAR1.g36423 |  |  |  | polar amino acid ABC transporter, inner membrane subunit |  |
|  |  | PfAR1.g36420 |  |  |  | L-pipecolate oxidase (1.5.3.7) |  |
|  |  | PfAR1.g36410 |  |  |  | Oxidoreductase (EC 1.1.1.-) |  |
|  |  | PfAR1.g36405 |  |  |  | Endoribonuclease L-PSP |  |
|  |  | PfAR1.g36400 |  |  |  | Cysteine synthase B (EC 2.5.1.47) |  |
