# Supplementary material for: Genetic and molecular characterization of multicomponent resistance of Pseudomonas against allicin
Source: Life Sci Alliance. 2020 Mar 31;3(5):e202000670. doi: 10.26508/lsa.202000670 (PMC7119367; doi:10.26508/lsa.202000670)
Supplement: Supplementary file 2 [file LSA-2020-00670_TableS2.docx]

**Table S2**: Syntenic regions in pseudomonads other than *Pf*AR-1, related to section “*In-silico analysis of the* Pf*AR-1 genome”*. The information in this Table is from the Pseudomonas.com database.

|  | **synt. regions** | **Location Name** | **isolation source** | **Assembly Accession** | **additional information** |
| --- | --- | --- | --- | --- | --- |
| **complete genomes** |  |  |  |  |  |
| *Pseudomonas brassicacearum* DF41 | 1 | Manitoba | canola root tip | GCF_000585995.1 |  |
| *Pseudomonas brassicacearum* LBUM300 | 1 | Canada: New Brunswick: Bouctouche | soil, Canada | GCF_001449085.1 |  |
| *Pseudomonas fluorescens* A506 | 1 | USA: California | pear tree leaf | GCF_000262325.2 |  |
| *Pseudomonas fluorescens* FW300-N2E3 | 1 | USA: Oak Ridge, TN | ground water from background well at DOE's FRC site at Oak Ridge | GCF_001307155.1 |  |
| *Pseudomonas fluorescens* Pt14 | 1 | India: Tinsukia, Assam | Rhizosphere soil | GCF_001747385.1 |  |
| *Pseudomonas frederiksbergensis* ERGS4:02 | 1 |  | glacial stream | GCF_001874645.1 |  |
| *Pseudomonas syringae* pv. tomato DC3000 | 1 |  |  | GCF_000007805.1 | host: *Solanum lycopersicum* (tomato) |
| *Pseudomonas trivialis* IHBB745 | 1 | India: Rong Tong, Lahual and Spiti | rhizosphere | GCF_001186335.1 | host: *Hippophae rhamnoides* (sea buckthorn) |
| **draft genomes** |  |  |  |  |  |
| *Pseudomonas aeruginosa* ATCC 33988 | 1 | USA: Ponca City, OK | fuel tank | GCF_000756575.1 |  |
| *Pseudomonas aeruginosa* ATCC 9027 | 1 | Australia: Sydney |  | GCF_001294675.1 | host: outer ear infection |
| *Pseudomonas aeruginosa* AZPAE12138 | 1 | USA: New York |  | GCF_000796525.1 | host: *homo sapiens* (human, cystic fibrosis) |
| *Pseudomonas aeruginosa* AZPAE14813 | 1 | India: Mumbai |  | GCF_000795085.1 | host: *homo sapiens* (human, urinary tract infection) |
| *Pseudomonas aeruginosa* AZPAE14898 | 1 | India: Chennai |  | GCF_000791035.1 | host: *homo sapiens* (human, respiratory tract infection ) |
| *Pseudomonas aeruginosa* AZPAE14947 | 1 | China: Beijing |  | GCF_000794045.1 | host: *homo sapiens* (human, urinary tract infection ) |
| *Pseudomonas aeruginosa* TRN6649 | 1 |  |  | GCF_001921175.1 | host: *homo sapiens* (human) |
| *Pseudomonas aeruginosa* WH-SGI-V-07643 | 1 | USA | Hospital | GCF_001452255.1 |  |
| *Pseudomonas amygdali* pv. *tabaci* ATCC 11528 | 1 |  |  | GCF_000145945.1 | This strain was sequenced independently in 3 different labs, with slight variations    host: *Nicotiana tabacum* (common tobacco) |
| *Pseudomonas amygdali* pv. *tabaci* ATCC 11528 | 1 |  |  | GCF_000159835.2 |  |
| *Pseudomonas amygdali* pv. *tabaci* ATCC 11528 | 1 |  |  | GCF_001006455.1 |  |
| *Pseudomonas brassicacearum* BS3663 | 1 |  |  | GCF_900103245.1 |  |
| *Pseudomonas brassicacearum* PA1G7 | 1 | France: Finistere | potato rhizosphere | GCF_000800585.1 | host: *Solanum tuberosum* (potato, soft-rot disease) |
| *Pseudomonas coronafaciens* pv. *porri* ICMP8961 | 1 | France |  | GCF_001400915.1 | host: *Allium ampeloprasum* (leek) |
| *Pseudomonas coronafaciens* pv. *porri* LMG 28495 | 1 | Belgium: Aarsele | plant | GCF_001275725.1 | host: *Allium ampeloprasum* (leek, leaf yellowing ) |
| *Pseudomonas coronafaciens* pv. *porri* LMG 28496 | 1 | Belgium: Menen | plant | GCF_001275735.1 | host: *Allium ampeloprasum* (leek) |
| *Pseudomonas fluorescens* ATCC 17400 | 1 | USA: California | hen's egg | GCF_000708695.2 |  |
| *Pseudomonas fluorescens* AU14917 | 1 | USA | sputum | GCF_000803005.1 | host: *homo sapiens* (human, cystic fibrosis) |
| *Pseudomonas fluorescens* EK007-7t-asp | 1 | not applicable | not applicable | GCF_001931665.1 |  |
| *Pseudomonas fluorescens* EK007-RG4 | 1 |  | phyllosphere | GCF_001902145.1 |  |
| *Pseudomonas fluorescens* ML11A | 1 |  | Skin mucus | GCF_001908925.1 | host: *Salvelinus fontinalis* (fish) |
| *Pseudomonas kilonensis* BS3780 | 1 |  |  | GCF_900105635.1 |  |
| *Pseudomonas lini* ZBG1 | 1 | France: Zellenberg | Soil | GCF_001238395.1 |  |
| *Pseudomonas mandelii* 36MFCvi1.1 | 1 |  |  | GCF_000381285.1 |  |
| *Pseudomonas marginalis* BS2952 | 1 |  |  | GCF_900105325.1 |  |
| *Pseudomonas orientalis* BS2775 | 1 |  |  | GCF_900105795.1 |  |
| *Pseudomonas orientalis* DSM 17489 | 1 | Lebanon | spring water | GCF_001439815.1 |  |
| *Pseudomonas plecoglossicida* TND35 | 1 | India: Tamilnadu, Ottanchathiram | soil | GCF_000764405.1 |  |
| *Pseudomonas putida* INSali382 | 1 | Portugal: Lisbon | Vegetable | GCF_001653615.1 |  |
| *Pseudomonas putida* JQ581 | 1 |  |  | GCF_001630725.1 |  |
| *Pseudomonas* sp. A214 | 1 |  |  | GCF_900156295.1 |  |
| *Pseudomonas* sp. C5pp | 1 | India: Mumbai | soil | GCF_000814065.1 |  |
| *Pseudomonas* sp. CFT9 | 1 | USA: Nyack River | hyporheic zone | GCF_000416255.1 |  |
| *Pseudomonas* sp. FSL W5-0203 | 1 |  | queso fresco | GCF_001896155.1 |  |
| *Pseudomonas* sp. GM55 | 1 |  |  | GCF_000282395.1 | host: *Populus deltoides* (eastern cottonwood) |
| *Pseudomonas* sp. GM67 | 1 | USA: Tennessee | endopshere | GCF_000282435.1 | host: *Populus deltoides* (eastern cottonwood) |
| *Pseudomonas* sp. ICMP 19500 | 1 | New Zealand | kiwi fruit | GCF_001467145.1 |  |
| *Pseudomonas* sp. QTF5 | 1 | China: Tuonamu area in Qiangtang basin | permafrost soil | GCF_000512695.2 |  |
| *Pseudomonas* sp. Root569 | 1 | Germany:Cologne | root | GCF_001427465.1 | host: *Arabidopsis thaliana* (tahle cress) |
| *Pseudomonas* sp. Root9 | 1 | Germany:Cologne | root | GCF_001429205.1 | host: *Arabidopsis thaliana* (tahle cress) |
| *Pseudomonas* sp. WCS374 | 1 | Netherlands: Flevoland | potato rhizosphere | GCF_000698295.1 | host: *Solanum tuberosum* (potato) |
| *Pseudomonas syringae* pv. *maculicola* 90_32 | 1 | USA: California |  | GCF_001293855.1 | host: *Brassica oleracea* |
| *Pseudomonas syringae* pv. *maculicola* ES4326 | 1 |  |  | GCF_000145845.1 |  |
| *Pseudomonas syringae* pv. tomato ICMP2844 | 1 | United Kingdom: Guernsey, Channel Islands |  | GCF_001401135.1 | host: *Solanum lycopersicum* (tomato) |
| *Pseudomonas syringae* pv. *maculicola* M4a | 1 | USA |  | GCF_001294305.1 | host: *Raphanus sativus* (radish) |
| *Pseudomonas syringae* pv. *persicae* isolate NCPPB 2254 | 1 |  |  | GCF_900235805.1 |  |
| *Pseudomonas syringae* pv. tomato PT23 | 1 |  |  | GCF_002024925.1 | host: tomato |
| *Pseudomonas umsongensis* UNC430CL58Col | 1 |  |  | GCF_000620285.1 |  |
| *Pseudomonas fluorescens* A3422A | 2 | USA: Alsea Valley Benton Co., OR | rhizosphere | GCF_002022335.1 |  |
| *Pseudomonas fluorescens* G2Y | 2 | USA: Linn Co., OR | rhizosphere | GCF_002022365.1 |  |
| *Pseudomonas fluorescens* TDH40 | 2 | USA: Philomath Benton Co., OR | rhizosphere | GCF_002022255.1 | host: Poa |
| *Pseudomonas salomonii* ICMP 14252 | 2 |  |  | GCF_900107155.1 |  |
| *Pseudomonas salomonii* LMG 22120 | 2 | France |  | GCF_001730645.1 | host: *Allium sativum* (garlic) |
| *Pseudomonas* sp. GM48 | 1 or 2 | USA: Tennessee | root | GCF_000282335.1 | host: *Populus deltoides* (eastern cottonwood) |
| *Pseudomonas syringae* pv. *maculicola* H7608 | 2 |  |  | GCF_001293925.1 | host: *Brassica campestris* (biennial turnip rape) |
| *Pseudomonas thivervalensis* LMG 21626 | 2 | France: Sexy-les-Bois | rhizoplane | GCF_001637285.1 | host: *Brassica napus* (rapeseed) |
