# Supplementary material for: Genetic and molecular characterization of multicomponent resistance of Pseudomonas against allicin
Source: Life Sci Alliance. 2020 Mar 31;3(5):e202000670. doi: 10.26508/lsa.202000670 (PMC7119367; doi:10.26508/lsa.202000670)
Supplement: Supplementary file 3 [file LSA-2020-00670_Supplemental_Data_1.pdf]

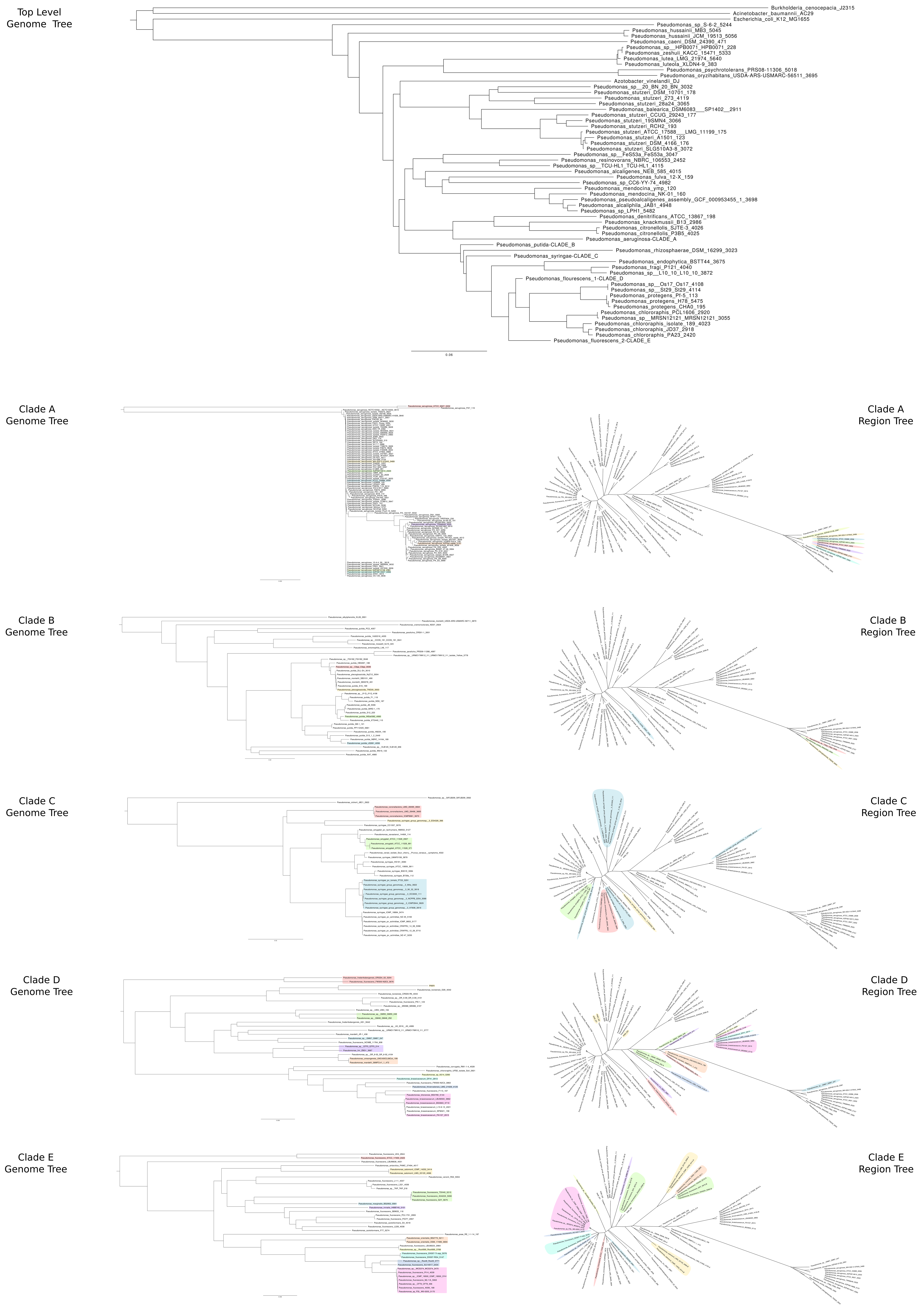
Top Level Genome Tree
Burkholderia_cenocepacia_J2315
Acinetobacter_baumannii_AC29
Escherichia_coli_K12_MG1655
Pseudomonas_sp_S-6-2_5244
Pseudomonas_hussainii_MB3_5045
Pseudomonas_hussainii_JCM_19513_5056
Pseudomonas_caeni_DSM_24390_471
Pseudomonas_sp__HPB0071_HPB0071_228
Pseudomonas_zeshuii_KACC_15471_5333
Pseudomonas_lutea_LMG_21974_5640
Pseudomonas_luteola_XLDN4-9_383
Pseudomonas_psychrotolerans_PRS08-11306_5018
Pseudomonas_oryzihabitans_USDA-ARS-USMARC-56511_3695
Azotobacter_vinelandii_DJ
Pseudomonas_sp__20_BN_20_BN_3032
Pseudomonas_stutzeri_DSM_10701_178
Pseudomonas_stutzeri_273_4119
Pseudomonas_stutzeri_28a24_3065
Pseudomonas_balearica_DSM6083___SP1402__2911
Pseudomonas_stutzeri_CCUG_29243_177
Pseudomonas_stutzeri_19SMN4_3066
Pseudomonas_stutzeri_RCH2_193
Pseudomonas_stutzeri_ATCC_17588___LMG_11199_175
Pseudomonas_stutzeri_A1501_123
Pseudomonas_stutzeri_DSM_4166_176
Pseudomonas_stutzeri_SLG510A3-8_3072
Pseudomonas_sp__FeS53a_FeS53a_3047
Pseudomonas_resinovorans_NBRC_106553_2452
Pseudomonas_sp__TCU-HL1_TCU-HL1_4115
Pseudomonas_alcaligenes_NEB_585_4015
Pseudomonas_fulva_12-X_159
Pseudomonas_sp_CC6-YY-74_4982
Pseudomonas_mendocina_ymp_120
Pseudomonas_mendocina_NK-01_160
Pseudomonas_pseudoalcaligenes_assembly_GCF_000953455_1_3698
Pseudomonas_alcaliphila_JAB1_4948
Pseudomonas_sp_LPH1_5482
Pseudomonas_denitrificans_ATCC_13867_198
Pseudomonas_knackmussii_B13_2986
Pseudomonas_citronellolis_SJTE-3_4026
Pseudomonas_citronellolis_P3B5_4025
Pseudomonas_aeruginosa-CLADE_A
Pseudomonas_putida-CLADE_B
Pseudomonas_rhizosphaerae_DSM_16299_3023
Pseudomonas_syringae-CLADE_C
Pseudomonas_endophytica_BSTT44_3675
Pseudomonas_fragi_P121_4040
Pseudomonas_sp__L10_10_L10_10_3872
Pseudomonas_flourescens_1-CLADE_D
Pseudomonas_sp__Os17_Os17_4108
Pseudomonas_sp__St29_St29_4114
Pseudomonas_protegens_Pf-5_113
Pseudomonas_protegens_H78_5475
Pseudomonas_protegens_CHA0_195
Pseudomonas_chlororaphis_PCL1606_2920
Pseudomonas_sp__MRSN12121_MRSN12121_3055
Pseudomonas_chlororaphis_isolate_189_4023
Pseudomonas_chlororaphis_JD37_2918
Pseudomonas_chlororaphis_PA23_2420
Pseudomonas_fluorescens_2-CLADE_E
0.06
Clade A Genome Tree
Clade A Region Tree
Clade B Genome Tree
Clade B Region Tree
Clade C Genome Tree
Clade C Region Tree
Clade D Genome Tree
Clade D Region Tree
Clade E Genome Tree
Clade E Region Tree
