# Supplementary material for: Genetic and molecular characterization of multicomponent resistance of Pseudomonas against allicin
Source: Life Sci Alliance. 2020 Mar 31;3(5):e202000670. doi: 10.26508/lsa.202000670 (PMC7119367; doi:10.26508/lsa.202000670)
Supplement: Supplementary file 4 [file LSA-2020-00670_TableS3.docx]

**Table S3:** List of bacteria used in this study for physiological or genetic experiments, supplemental to *materials and methods section*.

| **organism** | **reference** |
| --- | --- |
| *E. coli* K12 HB101 | (Boyer and Roulland-dussoix 1969) |
| *E. coli* K12 MegaX DH10B T1^R^ | Thermoscientific |
| *E. coli* K12 DH5α | (Hanahan 1983) |
| *E. coli* S17-1 λpir | (Simon et al. 1983) |
| *E. coli* MG1655 | (Blattner et al. 1997; Neidhardt and Curtiss 1996) |
| *E. coli* BW25113 | (Baba et al. 2006; Datsenko and Wanner 2000); Coli Genetic Stock Center Number CGSC#: 7636; Strain BW25113 |
| *E. coli* BW25113 *Δgor* | (Baba et al. 2006); Coli Genetic Stock Center Number CGSC#: 10569; Strain JW3467‑1 |
| *Pseudomonas fluorescens* Allicin resistant-1 (*Pf*AR-1) | this study |
| *Pseudomonas syringae* pv. *phaseolicola* strain 4612 | Robin E. Mitchell, Division of Horticulture and Processing, Department of Scientific and Industrial Research, Auckland, New Zealand |
| *Pseudomonas syringae* pv. *tomato* DC3000 | (Buell et al. 2003) |
| *Pseudomonas savastanoi* pv. *phaseolicola* strain 1448A | (Joardar et al. 2005) |
| *Pseudomonas salomonii* ICMP 14252 | (Gardan et al. 2002) |
