# Supplementary material for: Genetic and molecular characterization of multicomponent resistance of Pseudomonas against allicin
Source: Life Sci Alliance. 2020 Mar 31;3(5):e202000670. doi: 10.26508/lsa.202000670 (PMC7119367; doi:10.26508/lsa.202000670)
Supplement: Supplementary file 5 [file LSA-2020-00670_TableS4.docx]

**Table S4:** Plasmids used in this study, supplemental to *materials and methods section*.

| **plasmid** | **comments** | **source/reference** |
| --- | --- | --- |
| pSCR001 | contains the transposon IS-Ω-km/hah for transposon mutagenesis | (Giddens et al. 2007) |
| pRU1097 | promoter probe vector, broad host range, used to make genomic library from *Pf*AR-1 | (Karunakaran et al. 2005) |
| pJP2neo | modified version of pJP2 (Prell et al. 2002) source of neo-promoter for pJABO | Dr. Jürgen Prell, RWTH-Aachen, Unit of Sil Ecology, BioI |
| pBluescript I KS (-) | standard high copy cloning vector for *E. coli*. Source of MCS used to construct pJABO and pJABO5 | Stratagene Inc., La Jolla |
| pJABO | broad host range expression system, based on pRU1097 backbone | this study |
| pRS426 | yeast high copy expression vector (2µ ori, URA3). Used for construction of pJABO5. | (Mumberg et al. 1995) |
| pJABO5 | broad host range expression system, based on pRU1097 backbone | this study |
